# Adsorption of Heavy Metals on Alkali-Activated Zeolite Foams

**DOI:** 10.3390/ma17030685

**Published:** 2024-01-31

**Authors:** Eliška Svobodová, Zdeněk Tišler, Kateřina Peroutková, Kateřina Strejcová, Jan Abrham, Josef Šimek

**Affiliations:** Orlen UniCRE, a.s., Revoluční 1521/84, 400 01 Ústí nad Labem, Czech Republic; zdenek.tisler@orlenunicre.cz (Z.T.); katerina.peroutkova@orlenunicre.cz (K.P.); katerina.strejcova@orlenunicre.cz (K.S.); abrhamj@vscht.cz (J.A.); josef.simek@ujep.cz (J.Š.)

**Keywords:** adsorption, heavy metals, zeolite foam, alkali activation

## Abstract

Elevated concentrations of heavy metals in natural waters can cause significant ecological problems. It is therefore essential to ensure their removal from any water discharged into the environment immediately, especially in case of an accident, where there is a risk of releasing large quantities or high concentrations. The aim of this paper is to test a newly developed adsorbent for the removal of heavy metals from aqueous solutions—in particular, it is very fast adsorption, and thus efficiency, during clean-ups. The alkali-activated foamed zeolite adsorbent was laboratory-prepared and -tested in both batch and flow-through arrangements on single and multi-component solutions and compared with natural zeolite. The experimental setup for batch adsorption consisted of a set of samples and solutions containing iron, cobalt, manganese, zinc and nickel. The samples were put on a horizontal shaker with a 500 mg adsorbent loading in a 50 mL solution. The column adsorption experimental setup consisted of a glass column with an inside diameter of 15 mm and a bed length of 165 mm. A measured amount of each adsorbent was added to the column to achieve a filter fixed-bed height of 160 mm. The high efficiency of the tested adsorbent on various heavy metals was confirmed. The adsorbent has a high potential for use in decontamination processes, water protection and landscape revitalization. Due to its rapid precipitation and subsequent fixation of metal cations in the form of insoluble oxide or hydroxide, it can be used as an emergency adsorbent, the great advantage of which is its low production cost and natural origin.

## 1. Introduction

Almost all heavy metals are naturally present in waters, at least in trace amounts. The generic term, heavy metals, refers to elements that demonstrate metallic properties (transition metals, metalloids, lanthanides and actinides) and have a high specific gravity, 5.0 or greater [1]. The amount depends on geological and hydrogeological conditions, as the elements enter the water through the contact of water with soil and rocks. Nowadays, it is difficult to distinguish the sources of contamination as natural or anthropogenic, especially in the case of sediments, which may be another source of metal contamination of water as a result of remobilization processes. The main anthropogenic sources of metal and metalloid contamination of waters are wastewaters from mining and ore processing, metalworking industries, the photographic, textile and leather industries, agriculture and contact between water and pipes made of various materials [2,3].

For example, the usual concentrations of trace elements in lithogenic waters are 2–8 μg/dm^3^ for Cu, 5–50 μg/dm^3^ for Zn, 0.8–5 μg/dm^3^ for Ni and 10–50 μg/dm^3^ for Mn [4]. The toxicity of metals depends on the temperature, pH, cation strength and overall composition of the water. However, as their content increases, certain concentrations of certain forms of metals can cause various serious environmental problems [5].

Heavy metals persist in the environment, are biocumulative and are able to disrupt metabolic processes in living organisms, including humans.

One of the biggest producers of wastewater is mining, producing acid mine drainage, which usually contains Fe, Cu, Zn, Ni and sulphate ions [6], mostly because of oxidation of pyritic materials, which produce sulphuric acid and metal ions. These released substances, along with groundwater and air, react with surrounding rocks and create acidic water with elevated ion concentrations [7,8].

Acidity with elevated metal ion concentrations is especially harmful to the environment and this kind of water pollution. The problem associated with mine water is being addressed around the world. Contamination from mine water can persist long after mining has ceased. Pollution is not only associated with surface water, but also causes degradation of puddles and aquatic ecosystems and groundwater contamination. At the same time, it is not confined to the local area and has an impact on a more distant area if it is not dealt with at source and discharged into natural water streams [2].

It is therefore necessary to address this issue and to study the methods available for removing these elements from waters. A number of methods have been developed for this purpose, including adsorption. The adsorption method tends to be feasible, economically inexpensive and efficient, and is widely used in industry for the purification of gases and liquids, for the separation of gas mixtures and in the field of heterogeneous catalysis. In addition, it is usually more effective in removing low concentrations of contaminants. However, it depends on a number of conditions, including the choice of adsorbent, which must have optimum properties. Alternatively, for the removal of heavy metals, absorption combined with adsorption is more advantageous, because the contaminant will be firmly fixed in the sorption medium. The sorption medium can be of a whole range of materials—natural, waste or synthetic, with various types of additional treatments. Commonly in industry, adsorbents such as graphene, graphine oxide, activated carbon, carbon nanotubes, mesoporous silica, alumina, clay, chitosan, zeolites, red mud and magnetic adsorbents are used [9,10]. Also used are biomaterials, such as rice husk, sugar cane bagasse, wheat bran, coconut waste, orange peel, modified saw dust, modified eggshells, coffee residue, olive stones and various microorganisms [11].

One of the possibilities is inorganic porous materials, which are widely used in industry. They are used in adsorption and catalysis, and as supports for catalysts. In these application areas, their textural properties such as specific surface area, porosity and pore size distribution, their mechanical properties and chemical stability play a key role.

Another group of porous materials is alkali-activated materials, which have been studied in the past, mainly in the construction industry, for use as substitutes for concrete, ceramic materials and thermal insulation. However, their properties predispose them to much wider use in other sectors, especially in the chemical industry. They can be prepared using a variety of materials, including waste (ash, slag, sludge, tailings, waste from paper production, glass, etc.) and natural materials (metakaolin, kaolinite, volcanic ash, tuff, natural zeolites, etc.).

Alkali-activated zeolite foams represent a relatively new group of materials that, due to their structure containing macro- and meso-pores and, after treatment of the material, micro-pores. Such a structure positively influences the transport of the medium (gas, liquid) through the catalyst and ensures a lower specific gravity, thus reducing the weight of the catalytic bed. These materials have a heat resistance up to 600 °C. At higher temperatures, the zeolite structure breaks down, the acidity of the material decreases and an inert macroporous ceramic material is formed. Zeolite foams are prepared by alkaline activation of natural zeolite, followed by foaming of the mixture, resulting in a composite material composed of original zeolite structures connected into a solid foam matrix by a binder phase created by alkaline activation of the aluminosilicate raw material [12].

The aim of this study was to test the adsorption capacity of the prepared adsorbents—alkali-activated (AA) and alkali-activated foamed (AZF) versions of zeolite—and to compare them with the natural unmodified zeolite (KLI). Adsorption tests were performed in both batch and continuous flow arrangements on laboratory-prepared solutions with targeted concentrations of the metals of interest. The prepared adsorbents were extensively characterized using a number of techniques, such as X-ray diffraction (XRD), X-ray fluorescence (XRF), mercury porosimetry, N_2_ physisorption and scanning electron microscopy (SEM). Metal concentrations in aqueous solutions before and after the tests were determined via inductively coupled plasma optical emission spectrometry (ICP-OES) to evaluate the adsorption tests.

## 2. Materials and Methods

### 2.1. Materials

Natural zeolite ZEOCEM 50 in powdered form and in the fraction 560–850 μm was purchased from Zeocem, (Bystré, Slovakia). The chemical composition of this powdered material, which contains at least 85 wt % of clinoptilolite and other mineral components like clay, feldspar and mica (9, 4 and 2 wt %, respectively), is shown in Table 1.

Potassium hydroxide, magnesium oxide and hydrogen peroxide of analytical grade were supplied by Lach-Ner, s.r.o (Neratovice, Czech Republic). Na-water glass (sodium silicate) with silicate modulus 3.22 was supplied by Labar s.r.o (Ústí nad Labem, Czech Republic).

The following compounds were used to prepare the solutions for the adsorption tests:

- Iron(III) nitrate nonahydrate (Fe(NO_3_)_3_.9H_2_O) (Penta, s.r.o., Prague, Czech Republic)
- Manganese(II) nitrate tetrahydrate (Mn(NO_3_)_2_.4H_2_O) (Acros Organics B.V.B.A., Geel, Belgium)
- Copper(II) nitrate trihydrate (Cu(NO_3_)_2_.3H_2_O) (Acros Organics B.V.B.A., Geel, Belgium)
- Nickel(II) nitrate hexahydrate (Ni(NO_3_)_2_.6H_2_O) (Penta, s.r.o., Prague, Czech Republic)
- Zinc(II) nitrate hexahydrate (Zn(NO_3_)_2_.6H_2_O) (Lach-Ner, s.r.o., Neratovice, Czech Republic)

### 2.2. Methods

#### Synthesis of the Zeolite-Based Adsorbents

**Figure 1:**
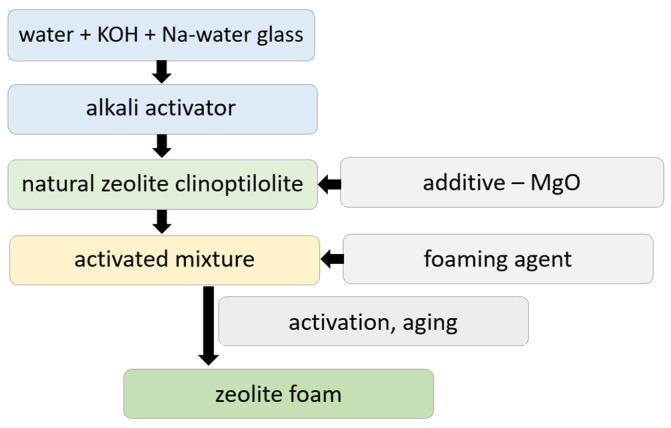
Scheme of the preparation of alkali-activated zeolite foam.

Alkaline-activated foamed zeolite was prepared by mixing natural zeolite with an alkaline activator and then adding a foaming agent, according to the scheme in Figure 1, the same method we used in our previous publications [13].

The raw material for the preparation was powdered natural zeolite ZEOCEM 50, which was weighed into a large plastic beaker in a quantity of 780 g. Next, 20 g of magnesium oxide powder was added, and the mixture was mixed by hand with a stirrer. An alkaline activator was prepared in a glass beaker by mixing 166 g of 40% KOH solution, 388 g of sodium silicate (Na-water glass) and 151 g of water. The alkaline activator thus prepared was added to the mixture of zeolite with MgO, and the mixture was homogenized. Before adding the foaming agent, the mixture was placed on a vibrating pad for 20 s to remove air bubbles from the mixture. A 30% H_2_O_2_ solution at 0.125% by weight (based on the activated zeolite mixture) was used as the foaming agent. After the addition of peroxide, the mixture was homogenized, placed back on the vibrating pad and transferred to plastic moulds, where it was allowed to stand for one hour, during which time foaming and setting occurred. In order to compare the effect of the presence of large pores in the adsorbent, the material (AA) was prepared in the same way but without the addition of foaming additive. After solidification, activation, i.e., heating to 50 °C for 48 h, was carried out, and then the solidified blocks of alkali-activated zeolite foam were transferred to airtight PE plastic bags and stored for 30 days at laboratory temperature for sample aging.

The obtained blocks of AZF were then crushed in a laboratory jaw crusher and sorted into individual grain size fractions using a laboratory sieve shaker (Retsch AS300, Retsch GmbH, Haan, Germany). The 560–850 μm fraction was used for adsorption tests.

### 2.3. Characterization

The structure of the crystalline phases of the materials was analyzed by powder X-ray diffraction using a D8 Advance ECO powder diffractometer (Bruker AXS GmbH, Karlsruhe, Germany) and evaluated using Diffrac.EVA software V4.1 from the same company with the Powder Diffraction File database (PDF 4 + 2018, International Centre for Diffraction Data).

The X-ray fluorescence method was used to determine the elemental composition using an S8 Tiger (Bruker AXS GmbH, Karlsruhe, Germany) equipped with an Rh cathode, and the measurement results were processed in Spectra plus software (Version 3).

Textural properties were analyzed by mercury porosimetry (Hg porosimetry) using AutoPore IV 9510, Micromeritics Instrument Corporation, Norcross, GA, USA, and N_2_ physisorption. N_2_ physisorption was performed at −196 °C using an Autosorb iQ instrument (Quantachrome Instruments, Boynton Beach, FL, USA). Pore size disstributions and specific surface area (BET) were determined by the N_2_ adsorption/desorption method. The samples were first dried. Drying was carried out under vacuum in glass cells at 110 °C for 16 h. Samples for mercury porosimetry were pre-dried in the same way.

The surface morphology and chemical compositions of the samples were determined using a scanning electron microscope with an energy-dispersive X-ray spectroscopy detector (EDX). Analyses were taken with a JSM-7500F (JEOL Ltd., Tokyo, Japan). The images were taken in high vacuum mode, using primary electrons backscattered from the sample surface and secondary electrons from the interaction between the sample and the electron beam to form the image. The acceleration voltage was 15.0 kV. The dried samples were gold-plated by the vapor deposition method prior to actual imaging.

An Inductively Coupled Plasma Optical Emission Spectrometer (ICP-OES) (Agilent Technologies, Inc., Santa Clara, CA, USA) was used to analyze concentrations of metals in aqueous solutions.

### 2.4. Adsorption Tests

The experimental setup for batch adsorption consisted of a set of samples and solutions put on a horizontal shaker Titramax 1000 (Heidolph Instruments GmbH & Co. KG, Schwabach, Germany) at 600 rpm for 24 h. A 500 mg adsorbent loading in 50 mL solution was used, optimized on the basis of previous tests. Samples were centrifuged before analysis at 6000 rpm for 20 min to remove adsorbent residue on the Centrifuge EBA200 (Andreas Hettich GmbH& Co. KG, Tuttlingen, Germany).

The column adsorption experimental setup consisted of a glass column with an inside diameter of 15 mm and a bed length of 165 mm. A measured amount of each adsorbent was added to the column to achieve a filter fixed-bed height of 160 mm. Solutions were prepared using a digital burette Titrette (BRAND GmbH + CO, Wertheim, Germany) were fed into the column using a membrane metering pump SIMDOS 10 (KNF FLODOS AG, Sursee, Switzerland) at a rate 25 mL/min. At the exit of the column, samples of the resulting solution were taken at predetermined time intervals into prepared beakers.

For each sample from all tests, the pH was measured using a FiveEasy Plus FP20 pH meter (Columbus, OH, USA) and the metal content of the sample was analyzed by ICP-EOS (mg/dm^3^). In addition to the selected heavy metals, the leachable elemental contents of the adsorbents (Na, K, Ca and Mg) and the effect of pH on their resulting concentrations were monitored.

## 3. Results and Discussion

### 3.1. Characterisation of Adsorbents

#### 3.1.1. XRF

The chemical composition was determined by XRF and shows the differences between the compositions of the studied samples. A very low Na content is documented for the KLI adsorbent. AZF and AA, on the other hand, have much higher content of Mg, K and Na. All these elements have been added during the synthesis and originate from an alkaline activator (Na+K) and from added MgO, which significantly improves the mechanical properties [14]. The synthesis also changes the Si/Al ratio, which increases compared with natural zeolite due to the alkaline activator containing Na-silicate. In Table 2, the chemical composition of the adsorbents determined by XRF can be observed.

#### 3.1.2. XRD

**Figure 2:**
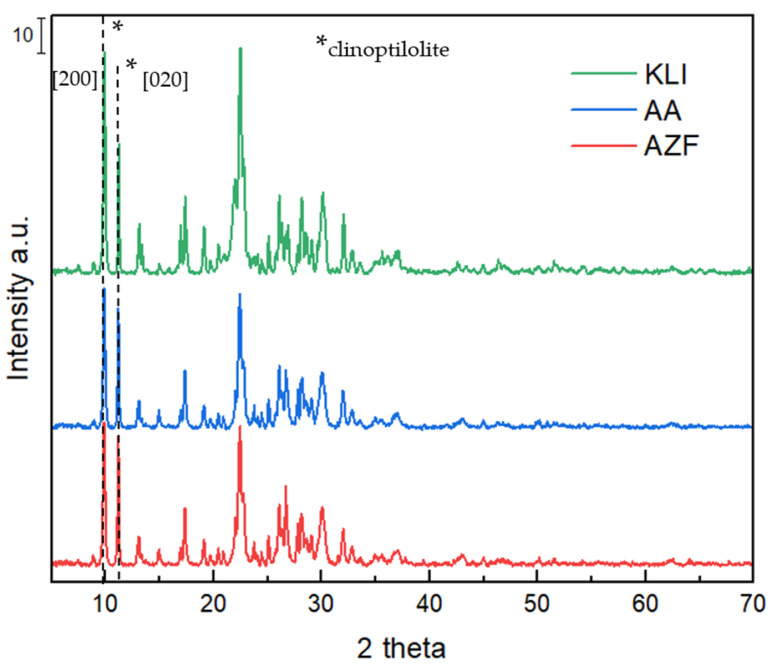
XRD diffractograms of adsorbents.

From the XRD diffractograms (Figure 2), it can be observed that all three adsorbents have the same basic raw material, namely, natural zeolite containing the main crystalline phase clinoptilolite, which is accompanied by minor crystalline phases of minerals from the group of micas, feldspars and clays (biotite, anorthite etc.). In contrast to KLI, the alkali-activated materials AA and AZF also contain a bonding N(K)-A-S-H phase, but this is amorphous and therefore not visible in the XRD diffractogram. Its presence is manifested by changes in the ratio of the intensities of the two most prominent reflections (2theta 9.9° and 11.2°) of clinoptilolite belonging to the planes [020] and [200] [14], which were caused by the higher electron density in the crystal plane due to a presence of Na and K cations [15]. Compared with the original natural zeolite (KLI), a decrease in intensities is observed due to partial disruption of the crystal structure by alkaline activation or its overlap with the amorphous N(K)-A-S-H phase [13].

### 3.2. Textural Properties

**Figure 3:**
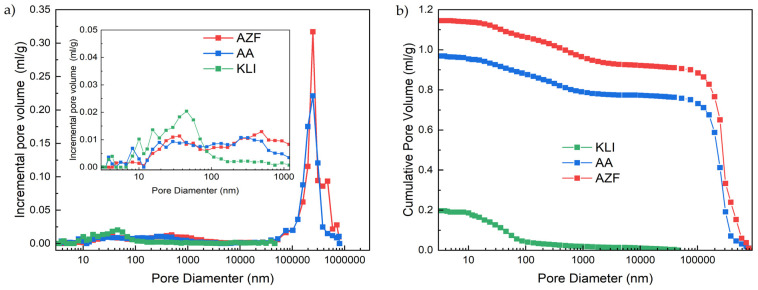
Pore size distribution (**a**) and Cumulative pore volume (**b**).

Textural properties were evaluated by N_2_ physisorption (micro- and mesopores) and Hg porosimetry (meso- and macropores). The combination of these two methods allowed the determination of the pore size distribution of the materials and showed a wide range of pore sizes in the materials used. The Hg porosimetry results (Figure 3) showed that all samples contained mesopores (3–50 nm) and macropores (>50 nm). A higher proportion of macropores with a size of hundreds of micrometers is evident in the AZF material than in the AA and KLI materials. Porosimetry has certain limitations in regard to pores with a size of several hundreds of microns. Thus, there may be larger pores in AZF, but the method has not detected them.

The results of physisorption measurements (Table 3) showed that the samples did not contain micropores (0–2 nm) but mostly mesopores. Although zeolites are generally microporous materials, natural zeolite does exhibits barely any microporosity because the micropores are not accessible and are blocked by other components, fragments formed during their formation, cations, etc. [13]. Due to their size, the macropores present are not detected by N_2_ physisorption because their size is in the order of hundreds of nm to hundreds of micrometers, as shown by the Hg porosimetry results (Figure 3). The volume of each pore type was evaluated using the Non-Local Density Functional Theory (NLDFT) method.

The specific surface area of the samples was evaluated using the BET (Brunauer-Emmet-Teller) method, calculated using the BET equation in the linear range of 0.05–0.30 p/p0 (relative pressure; more precisely, the pressure of the adsorbate relative to its saturation pressure). The KLI adsorbent sample showed the largest specific surface area. The specific surface area value of activated materials was lower compared with natural zeolite due to the partial blocking of the pores by the alkali activator (area around 5–30 nm, Figure 3). The properties of the adsorbents AA and AZF are almost identical, as both are prepared by alkaline activation, but AZF differs mainly due to the presence of large macropores formed by the foaming of the alkaline-activated mixture with gaseous O_2_, which was formed by the decomposition of H_2_O_2_ in a strongly alkaline medium (Equation (1)).

2 *H*_2_*O*_2_ (*l*) → 2 *H*_2_*O* (*l*) + *O*_2_ (*g*) (1)

All macropores may not be detected by Hg porosimetry due to the theoretical size limit of approx. 360 µm (published in Micromeritics literature). These differences in macropore content are illustrated by the bulk density of AZF, which was lowest (710 g/dm^3^), while the non-foamed material (AA) had a bulk density of 820 g/dm^3^, and natural zeolite (KLI), 950 g/dm^3^.

**Figure 4:**
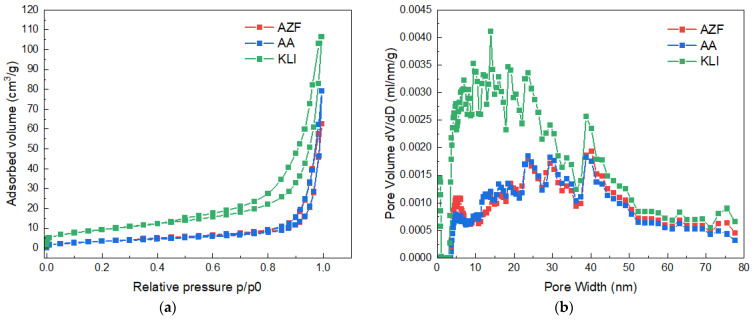
Adsorption/desorption N_2_ isotherm at −196 °C (**a**) and Pore size distribution (**b**).

The adsorption isotherms from the physisorption measurements can be observed in Figure 4. The shape of the curve shows both N_2_ adsorption and reverse desorption. All samples within this work exhibited a type IV(a) isotherm, which is typical of mesoporous materials and forms a so-called hysteresis loop according to IUPAC. The formation of the hysteresis loop was due to the condensation of N_2_ in the mesopores, thus again proving the existence of mesopores in the samples [16,17]. The shape of the loop (hysteresis type) indicates the probable shape of the pores. For all the adsorbent samples measured, the closest type was H3 [18,19].

#### SEM

**Figure 5:**
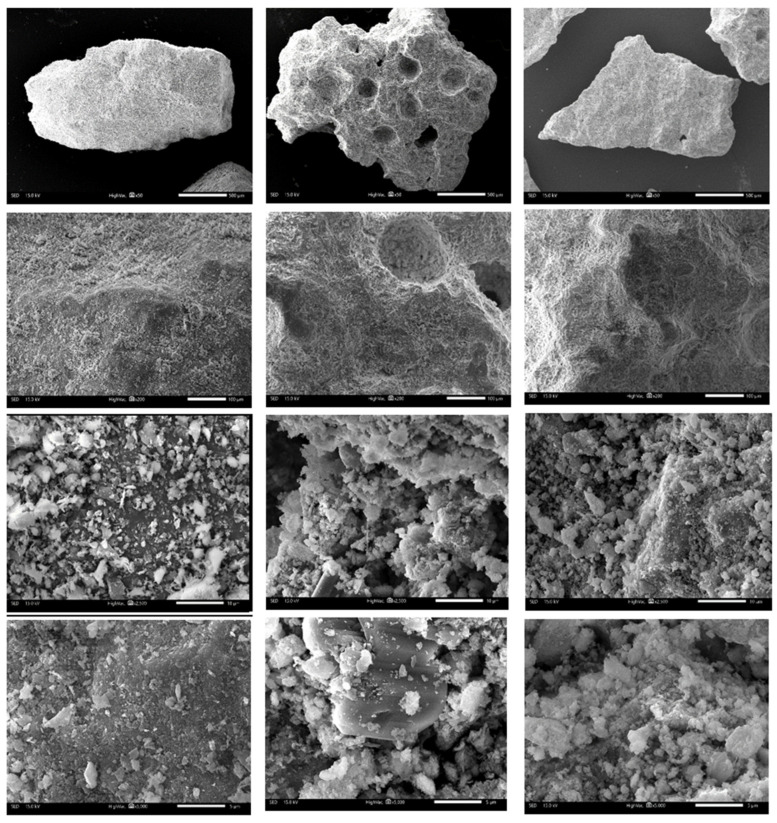
Scanning electron microscope images of the tested adsorbents: KLI (first column), AZF (second column) and AA (third column)—magnification 50× (first row), 200× (second row), 2500× (third row) and 5000× (fourth row).

The structure of the adsorbents can be observed in the SEM images (Figure 5).

The KLI mineral sample showed a quite compact structure, in which macro-pores occasionally appeared, and, in higher magnifications, it can be observed that clinoptilolite is a plate-shaped material. Its finer particles form into bigger aggregates.

Images of the AZF sample showed the effect of foaming, which leads to the formation of large macropores and bubble-like hollows in the material.

**Figure 6:**
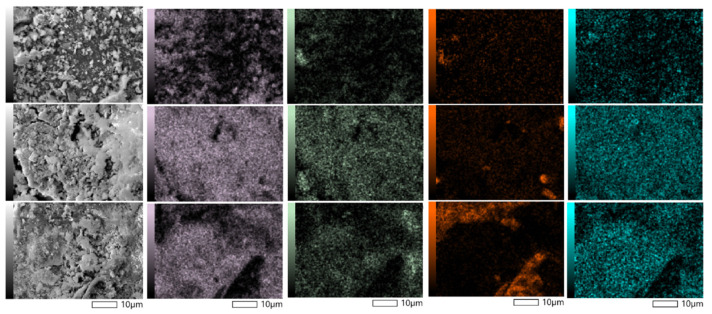
EDX mapping analysis of adsorbents: KLI (first row), AZF (second row) and AA (third row) and elements of interest: Si (mauve), Al (green), Na (orange), K (blue).

In contrast to KLI, AA and AZF showed crystalline structures formed by the reaction of the alkaline activator (see Figure 6 SEM-EDX). EDX mapping showed that the potassium distribution is more homogeneous than the sodium distribution. This suggests that potassium was mainly involved in the formation of the N(K)-A-S-H bonding matrix, while sodium formed I structures independently, which usually leads to the formation of undesirable efflorescence in alkaline materials and geopolymers. This process is particularly undesirable when used as a substitute for concrete, where air humidity and the presence of CO_2_ leads to the formation of unsightly Na carbonates on the surface of the materials. In the case of AA and AZF adsorbents, on the other hand, alkalis were used in the process of adsorption of heavy metal cations.

The prepared adsorption materials were thoroughly characterized using several methods. In particular, attention was paid to the comparison of alkali-activated samples (foamed (AZF) and non-foamed (AA)) with natural zeolite (KLI). The greatest differences were observed in textural properties, where AZF in particular stood out for its low bulk density, which was 75% of KLI. Alkali-activated materials also differed from natural zeolite by having significantly higher amounts of alkalis (Na and K), which contributed to better adsorption properties, as evidenced by subsequent adsorption tests. The specific surface areas of both alkali-activated samples are very similar (around 13 m^2^/g) and slightly lower that natural zeolite KLI (34 m^2^/g).

### 3.3. Laboratory Adsorption Experiments—Batch

First, trial tests were carried out for a better understanding of the adsorption mechanism and to find the time required for the equilibrium to settle. Series of solutions with increasing concentration were prepared immediately before each test (c_metal_: 0–2000 mg/dm^3^). Based on their evaluation, a period of 24 h was confirmed, which is commonly used [20,21,22,23]. The adsorption efficiencies of the adsorbents in the batch experiments were calculated according to Equation (2):

*qe* = (*c*0 − *c*) × *V*/*m* (2)

where *qe* is the amount of metal adsorbed (mg/g), *c*0 is the initial concentration of the metal of interest in the solution (mg/dm^3^), *c* is the final concentration of the metal of interest in the solution (mg/dm^3^), *V* is the volume of the solution (dm^3^) and *m* is the mass of the adsorbent (mg).

**Figure 7:**
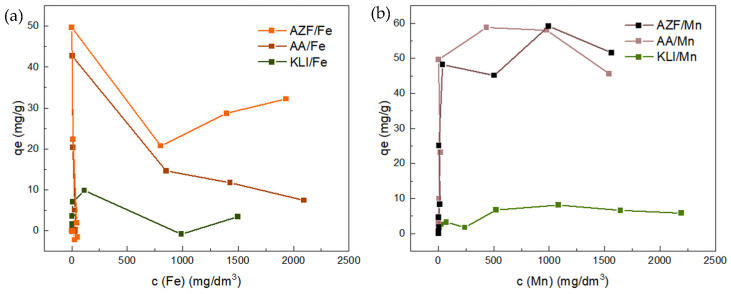
Batch adsorption experiments with Fe (**a**) and Mn (**b**) solution.

As shown in Figure 7, the removal efficiencies of three different adsorbents differed for Fe and Mn. The foamed adsorbent AZF had the highest overall efficiency in the removal of both Fe and Mn.

It can be seen how, with increasing concentrations of Fe/Mn cations in the solution, there was no longer enough alkali present in the adsorbent to precipitate all the cation into the insoluble form. This breakthrough occurred with the addition of around 500 to 1000 mg/dm^3^ solution. With the addition of higher-concentration solutions, all of the cation was no longer fixed and some remained in the solution.

**Figure 8:**
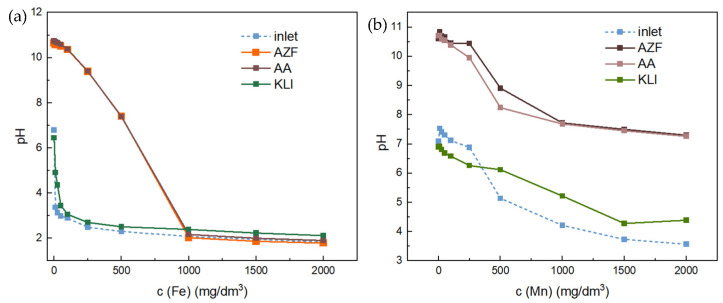
Solution pH after batch adsorption experiments: Fe (**a**) and Mn (**b**).

For deeper understanding, the initial as well as final pH of individual tests are depicted in Figure 8.

In the case of natural zeolite (KLI), there was no significant change in pH, i.e., the final pH of the solutions followed the pH of the initial solution. In contrast, when using the alkali-activated materials AA and AZF, the pH changed significantly due to the release of alkali from the adsorbent. In the case of Fe, the decrease was steeper than that of Mn because the pH of the initial Fe solutions was lower than that of the solutions containing Mn. From a concentration of 1000 mg Fe/dm^3^, the pH of the solution was already replicated, indicating complete neutralization of the alkali from the adsorbent. When a solution with a higher concentration was added, although adsorption, i.e., the elimination of Fe into an insoluble form, occurred initially, the excess acidic solution resulted in a re-release into solution until equilibrium was established. This phenomenon was well evident in the continuous flow tests. In contrast, the pH for Mn adsorption remained consistently high enough to allow Mn to precipitate further, the pH curve being above that of the initial solutions.

**Figure 9:**
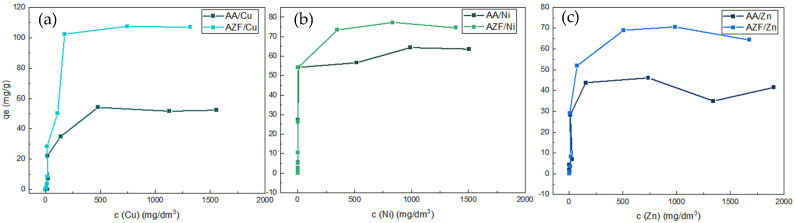
Adsorption experiments with Cu (**a**), Ni (**b**) and Zn (**c**) cation (batch tests).

**Figure 10:**
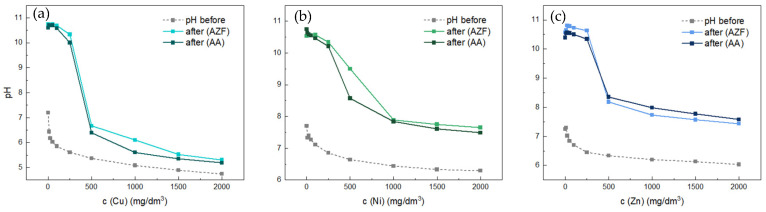
pH of residual solutions after adsorption tests: Cu (**a**), Ni (**b**) and Zn (**c**).

The adsorbents AA and AZF were further tested for the adsorption of Cu, Ni and Zn. As can be seen in the Figure 9, AZF was more efficient in all three experiments. The order of removal efficiencies for the three metals was Cu > Zn > Ni. As shown in Figure 9 and Figure 10, the adsorption and pH of the solutions were very similar to the previous cases. The original Cu, Ni and Zn solutions had similarly high pH as the Mn solutions. The steep drop in pH around a concentration of 500 mg/dm^3^ again indicated depletion of alkali from the adsorbent. All final concentrations can be found in Table A1.

Cu solution changed pH less than other two metal solutions, especially in case of higher concentrations. Both adsorbents affected the pH of the solutions very similarly, due to similar alkali content. For all three metals, the pH was significantly higher at low concentrations. In the case of Cu, there was a sharp drop in pH at the 500 mg/dm^3^ concentration point, which then gradually decreased and was close to the initial value at the 2000 mg/dm^3^ concentration, which could be related to the higher amount of metal removed than for the other two. In the case of the other two metals, the pH values were very similar. A sharp decrease occurred for Zn at a concentration of 500 mg/dm^3^ and for Ni at a concentration of 1000 mg/dm^3^, after which, the pH values remained considerably higher than the initial ones.

### 3.4. Laboratory Adsorption Experiments—Continuous Flow

Based on the results of the batch adsorption tests, where the best results were obtained using AZF adsorbent, the adsorption properties of AZF adsorbent were further tested in a continuous flow arrangement with Fe (c: 200 mg/L) and with a mixture of Fe, Mn, Cu, Ni and Zn (c: 200 mg/L = 40 mg/L of each metal ion).

The optimum flow rate with respect to complete wetting of the adsorbent and elimination of bubble formation, given the selected adsorbent fraction (580–1120 μm) and the column dimensions (15 × 165 mm), was chosen to be 25 mL/min.

Previous tests indicated that the main mechanism was the precipitation of cations on the adsorbent surface due to the gradual diffusion of alkalis (especially Na and K) from the structure of alkaline-activated materials; thus, for the cations used, it was the formation of hydroxides, with the foam structure of AZF contributing to the deposition of precipitation products due to its macropore content.

**Figure 11:**
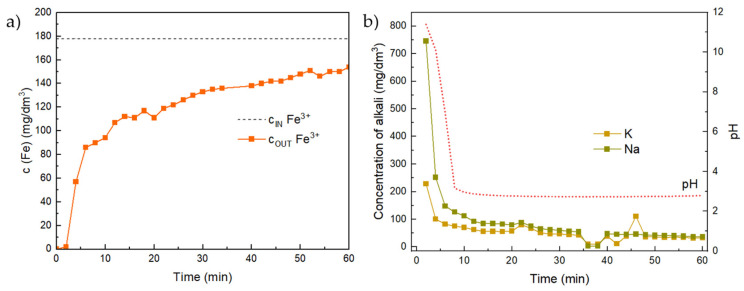
Continuous flow experiment with Fe solution: (**a**) concentration of Fe on the outlet and (**b**) pH and concentrations of alkali released into the solution as a function of time.

In Figure 11, we can observe the gradual adsorption and rerelease over time and the effect of pH on the adsorption process. So-called breakthrough curves are usually used to describe the adsorption process during flow tests. An important point here is the breakthrough point, which marks the point at which the adsorbent is no longer able to capture the contaminant perfectly and when the concentration of the contaminant in the solution at the outlet of the column increases. Another important concept here is the saturation point, where the concentration of the contaminant in the solution at the exit of the column is the same as its concentration in the initial solution at the entrance of the column [24].

The course of adsorption of Fe itself is shown in Figure 11. The graph shows that in the first moments, all Fe was removed, but after a relatively short time (around 7 min), the concentration began to increase, and the pH began to decrease, simultaneously.

The following cations were selected for laboratory tests: Ni^2+^, Cu^2+^, Fe^3+^, Mn^2+^ and Zn^2+^. They were chosen because they are the most common contaminants from ore processing and mining, electroplating processes, anticorrosive treatment of metals, photographic, textile and leather industries, agriculture, contact of water with pipes made of various materials, etc.

**Figure 12:**
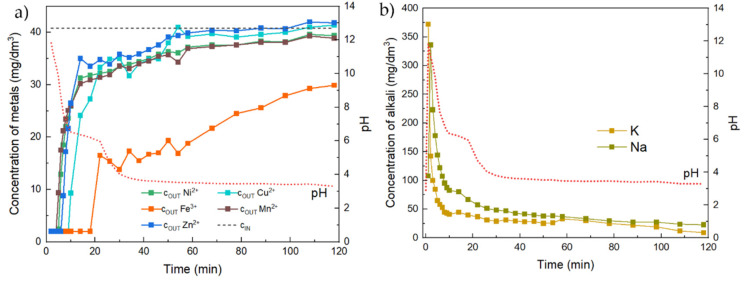
Amount of metal cations adsorbed on AZF adsorbent in a flow arrangement (**a**) and pH evolution as a function of time (**b**).

In can be seen that, within the first four minutes of the test, the cations were adsorbed and remained trapped in the column on the adsorbent (Figure 12). Around the fifth minute, metal cation concentrations started to increase due to the decrease in pH. Due to the depletion of alkali, the ability of the adsorbent to bind/fix the cation rather rapidly decreased. Up to about 20 min, the pH was still high enough for precipitation, but then it dropped to the level of the input. Some adsorption still occurred (the output was lower than the input), but here the excluded hydroxides could also play a role, as they could be porous and adsorption could take place on them as well. That happened at a different time for each cation, i.e., the time when the breakthrough point for that cation occurred. The earliest was observed for Mn at the fourth minute, followed by Ni from the fifth minute and Zn from the sixth minute. From the ninth minute onwards, the breakthrough point for Cu was observed, and lastly, from the 18th minute onwards, the breakthrough point for Fe was observed. The fact that Fe was last suggested the stabilization of Fe hydroxide by other hydroxides. In the previous test (Figure 11) where only Fe was used, it was evident that the breakthrough occurred after 2 min. In this test, the breakthrough points of individual cations were as follows: Mn at 4 min, Ni at 5 min, Zn at 7 min, Cu at 10 min and Fe at 18 min.

The reason for the quick removal of metals was precipitation by the alkalis contained in the adsorbent. The metals started to be released back due to the fact that all the alkalis had already been used up from the adsorbent and then the acidic solution leached the precipitated metals in the corresponding hydroxides back to solution. This was illustrated by the pH curve (Figure 12b), which reaches values up to pH = 12 at the maximum; then the pH value dropped significantly to neutral after a few minutes (approx. 10 min after the start of the test). This was followed by a gradual decrease, which accelerated again after the 20th minute of the test, and the pH of the output solution gradually decreased to the input values. Around the sixtieth minute of the continuous flow test, the concentration of most cations approached the initial concentration, i.e., the saturation point, and the pH of the solution approached the initial value. In contrast to solutions containing Mn, Cu, Zn and Ni, the Fe^3+^ solution is not stable at neutral pH. For this reason, Fe adsorption still occurred (between the 10th and 20th minute), while the release of insoluble Fe species no longer occurred because the pH dropped below the value at which they precipitate.

The pH of the solution was monitored during the tests to evaluate its effect on the release of alkali into the solution, which in turn affected the pH of the solution. The following Figure 12b shows the leaching of alkali from the adsorbent into solution, including the effect of pH as a function of time.

The increase in pH was caused by the leaching of K^+^, Na^+^, Ca^2+^ and Mg^2+^ ions. The leaching of Ca^2+^ and Mg^2+^ ions was visible only for Fe^3+^ ion adsorption. For the other tested solutions (Mn and Zn), the concentration of Ca and Mg remained practically unchanged and very low (up to 10 mg/dm^3^). This is probably due to the very low pH of the initial Fe solution (pH 2.3). In the case of the other metals (pH 6–6.4), the pH of the solutions was not low enough to leach Ca and Mg from the structure of the alkali-activated material. The highest concentrations were measured in the case of Na leaching because it is the most easily leached from the adsorbent structure. During the adsorption process, diffusion of alkalis, especially Na and K, from the adsorbent to the surface also occurred. The metal cation is then precipitated to the corresponding hydroxide, according to the model Equation (3):

M^x+^ (aq) + x OH^−^ (aq) → M(OH)_x_ (s) (3)

where *m* stands for metal (Fe, Ni, Mn, Cu or Zn).

Based on the obtained data, it can be concluded that the primary functional process in all adsorption tests performed was, in the case of alkali-activated adsorbents, the precipitation of cation on the surface and inside the adsorbent due to the gradual diffusion of alkali, especially Na and K from the adsorbent grain.

Since the tested materials showed very good adsorption properties, they have a high potential for use in decontamination processes in landscape revitalization, especially in the treatment of acid mine waters containing heavy metals. Due to their ability to rapidly precipitate and subsequently fix the metal cation in the form of insoluble oxide or hydroxide, they can be used as emergency adsorbents.

**Figure 13:**
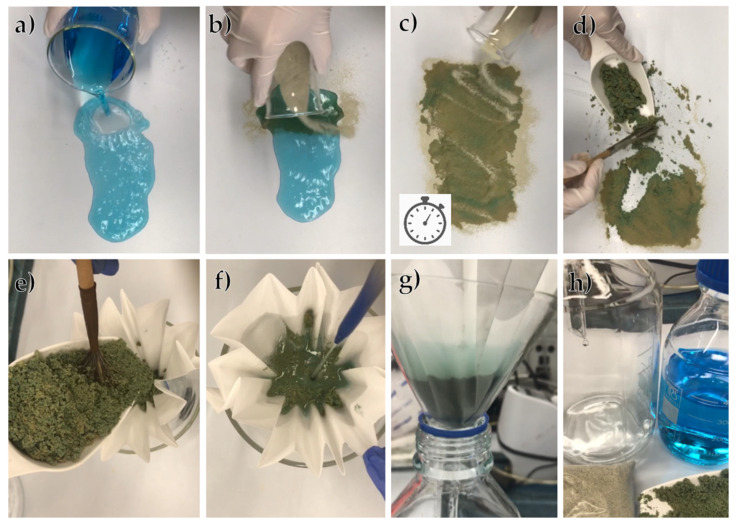
A demonstration of a spillage accident of Cu(NO_3_)_2_ solution (**a**), covering the spillage with AZF adsorbent and letting it sit for 10 min (**b**,**c**), transfer of the used adsorbent to filtration (**d**,**e**) and subsequent rinsing with distilled water (**f**,**g**). Comparison of initial solution and rinse water and used versus fresh adsorbent (**h**).

A demonstration of quick pollutant adsorption and immobilization can be seen in Figure 13, using Cu(NO_3_)_2_ solution (c = 10 g/dm^3^). It can be seen that, after flushing with demineralized water, Cu stayed fixed in the adsorbent and the water was not contaminated, as verified by ICP analysis.

Natural and waste inorganic adsorbents such as natural zeolite (clinoptilolite), kaolinite, bentonite and fly ash usually have very small adsorption capacity for heavy metals (Table 4). By modifying them (e.g., alkaline activation), the adsorption capacity increases significantly, which is due to structural changes on the one hand (formation of the N(K)-A-S-H binder matrix) and, on the other, the presence of free alkalis in the material, which, after release, cause the precipitation of heavy metal cations. This affects subsequent comparisons, as each type of alkali-activated material requires a different SiO_2_/Me_2_O (Me = Na or K) molar ratio at synthesis, which indicates the amount of alkali in the material. While the molar ratio for AA/AZF with SiO_2_/Me_2_O = 7.5 for geopolymers, this ratio is typically in the range of 3 to 5, i.e., the geopolymer material contains more alkalis that can be released and promotes the precipitation of heavy metals [25].

## 4. Conclusions

Adsorption is an important method for treatment of heavy metal-contaminated water, so the search for new materials with better adsorption characteristics is relevant to improve process efficiency. Results of this study suggest that these novel materials, whose use in adsorption processes has not yet been sufficiently studied, have considerable potential, particularly in the treatment of gases and waste, sewage and drinking water. The adsorption tests showed that the best properties of the prepared adsorbents had AZF, i.e., alkaline-activated foamed adsorbent based on natural zeolite. Due to its large pore volume, it allows better contact with the cations in aqueous solution and has a lower bulk density, and thus does not weigh down on the column apparatus too much. Also, its cost and difficulty of preparation are relatively low. As for the contaminants, the adsorption of Fe and Cu was the best for AZF, in terms of adsorbed amount. In the case of Mn adsorption, equilibrium was reached the most quickly, but the adsorbed amount was small compared to those of Fe and Zn. Furthermore, it was possible to observe a very low efficiency of the KLI adsorbent, in which, in contrast to the alkali-activated adsorbents, only ion exchange took place. As the tested materials showed very good adsorption properties, they have a high potential for use in decontamination processes in landscape revitalization, especially in the treatment of acid mine waters containing heavy metals. Due to their ability to rapidly precipitate and subsequently fix the metal cation in the insoluble form (hydroxide etc.), they can be used as emergency adsorbents. Furthermore, the studied adsorption materials can be modified in various ways, in terms of enhancing pore structure, alkali activation, etc., which adds considerably to their application potential. To investigate the competitive effect of the different metals on removal efficiency, more contaminant systems will be further studied.

## Figures and Tables

**Table 1:** Chemical composition of natural zeolite ZEOCEM 50.

Sample	Chemical Composition (Wt %)								Molar Ratio
	SiO_2_	Al_2_O_3_	K_2_O	Fe_2_O_3_	CaO	Na_2_O	MgO	TiO_2_	Si/Al
ZEOCEM 50	74.20	12.20	4.70	2.19	4.70	0.24	0.72	0.27	5.07

**Table 2:** Results of XRF analysis of adsorbents.

Sample	Wt %									Molar Ratio	
	SiO_2_	Al_2_O_3_	K_2_O	Fe_2_O_3_	CaO	Na_2_O	MgO	TiO_2_	SUM	SiO_2_/Al_2_O_3_	Si/Al
AZF	68.80	10.00	8.63	1.19	2.72	5.12	3.19	0.16	99.81	11.68	5.84
AA	69.10	9.86	8.55	1.25	2.83	4.96	3.07	0.17	99.79	11.89	5.95
KLI	75.30	12.60	4.26	1.91	4.28	0.30	0.78	0.23	99.66	10.14	5.07

**Table 3:** Textural properties of adsorbents.

Parameter	AA	AZF	KLI
Surface area BET (m^2^/g) *	12.8	13.6	24.5
Total pore volume (cm^3^/g) *	0.12	0.10	0.16
Mesopore volume (cm^3^/g) *	0.06	0.06	0.11
Total intrusion volume (mL/g) **	0.969	1.145	0.487

* measured by N2 physisorption. ** measured by Hg porosimetry.

**Table 4:** Comparation of the results with published analogs by adsorption capacity.

Adsorbent	Metal Ion	qe (mg/g)	Reference
AZF	Cu^2+^	107.5	Present Study
	Fe^3+^	49.7	Present Study
	Ni^2+^	77.3	Present Study
	Mn^2+^	59.2	Present Study
	Zn^2+^	70.6	Present Study
AA	Cu^2+^	54.1	Present Study
	Fe^3+^	42.7	Present Study
	Ni^2+^	64.4	Present Study
	Mn^2+^	58.8	Present Study
	Zn^2+^	46.1	Present Study
Natural zeolite	Cu^2+^	20.0	[26]
	Ni^2+^	8.7	[27]
	Zn^2+^	13.4	[28]
Fly ash	Cu^2+^	0.8	[29]
	Ni^2+^	9.0	[30]
	Zn^2+^	6.5	[30]
Kaolinite	Zn^2+^	1.3	[31]
Bentonite	Cu^2+^	4.5	[32]
Geopolymer	Cu^2+^	40.9	[33]
	Zn^2+^	74.5	[34]
	Ni^2+^	42.6	[34]
	Mn^2+^	72.3	[35]

## Data Availability

Data are contained within the article.

## Appendix A

**Table A1:** Final concentrations of each pollutant during batch experiments.

c (Initial) (mg/dm^3^)	c (Final) (mg/dm^3^)											
	Fe			Mn			Cu		Ni		Zn	
	AZF	AA	KLI	AZF	AA	KLI	AZF	AA	AZF	AA	AZF	AA
0	3	<2	<2	<2	<2	<2	<2	<2	<2	<2	<2	<2
10	10	9	<2	<2	<2	<2	6	8	<2	<2	2	<2
25	22	24	<2	<2	7	7	10	11	<2	<2	10	<2
50	33	53	<2	3	3	25	13	18	<2	<2	11	<2
100	29	44	11	5	15	72	22	25	<2	<2	16	29
250	10	12	113	19	6	239	15	18	<2	<2	6	12
500	4	5	380	8	39	420	112	139	<2	9	74	155
1000	852	803	988	434	505	984	175	477	349	517	507	736
1500	1428	1396	1498	978	996	1644	748	1129	835	991	987	1343
2000	1994	1931	1981	1542	1561	2191	1318	1557	1388	1507	1675	1903
